# Impact of metformin on cardiovascular and kidney outcome based on kidney function status in type 2 diabetic patients: a multicentric, retrospective cohort study

**DOI:** 10.1038/s41598-024-52078-4

**Published:** 2024-01-24

**Authors:** Yongjin Yi, Eun-Jeong Kwon, Giae Yun, Seokwoo Park, Jong Cheol Jeong, Ki Young Na, Ho Jun Chin, Sooyoung Yoo, Seok Kim, Tae Jung Oh, Sejoong Kim, Chang Hee Jung, Hajeong Lee

**Affiliations:** 1 Department of Internal Medicine, Dankook University College of Medicine, Cheonan-si, Republic of Korea; 2 Division of Nephology, Department of Internal Medicine, Seoul National University Bundang Hospital, Seongnam-si, Republic of Korea; 3 Healthcare ICT Research Center, Seoul National University Bundang Hospital, Seongnam-si, Republic of Korea; 4 Department of Internal Medicine, Seoul National University College of Medicine, Seoul, Republic of Korea; 5 Center for Artificial Intelligence in Healthcare, Seoul National University Bundang Hospital, Seongnam-si, Republic of Korea; 6 Department of Endocrinology and Metabolism, Asan Medical Center, University of Ulsan College of Medicine, Seoul, Korea; 7 Department of Internal Medicine, Seoul National University College of Medicine, Seoul, Korea; 8 Department of Internal Medicine, Seoul National University Hospital, Seoul, Korea

**Keywords:** Diabetes, Chronic kidney disease

## Abstract

Metformin is the primary treatment for type 2 diabetes mellitus (T2DM) due to its effectiveness in improving clinical outcomes in patients with preserved renal function, however, the evidence on the effectiveness of metformin in various renal functions is lacking. We performed a retrospective, multicenter, observational study used data of patients with T2DM obtained from three tertiary hospitals’ databases. Patients given metformin within run-in periods and with at least one additional prescription formed the metformin cohort. A control cohort comprised those prescribed oral hypoglycemic agents other than metformin and never subsequently received a metformin prescription within observation period. For patients without diabetic nephropathy (DN), the outcomes included events of DN, major adverse cardiovascular events (MACE), and major adverse kidney events (MAKE). After 1:1 propensity matching, 1994 individuals each were selected for the metformin and control cohorts among T2DM patients without baseline DN. The incidence rate ratios (IRR) for DN, MACEs, and MAKEs between cohorts were 1.06 (95% CI 0.96–1.17), 0.76 (0.64–0.92), and 0.45 (0.33–0.62), respectively. In cohorts with renal function of CKD 3A, 3B, and 4, summarized IRRs of MACEs and MAKEs were 0.70 (0.57–0.87) and 0.39 (0.35–0.43) in CKD 3A, 0.83 (0.74–0.93) and 0.44 (0.40–0.48) in CKD 3B, and 0.71 (0.60–0.85) and 0.45 (0.39–0.51) in CKD 4. Our research indicates that metformin use in T2DM patients across various renal functions consistently correlates with a decreased risk of overt DN, MACE, and MAKE.

## Introduction

Diabetic nephropathy (DN) is a common complication that occurs in approximately 40% of type 2 diabetes mellitus (T2DM) patients that can lead to deterioration of renal function and end-stage kidney disease (ESKD)^1,2^. DN in diabetic patients not only affects renal outcomes, but also increases risks of cardiovascular complications and mortality^3^. To date, only a few classes of antidiabetic drugs could improve renal outcomes in large-scale randomized clinical trials^4–7^. Most clinical trials on newer therapeutic agents have been designed for cardiovascular outcome or mortality, with relatively small number of studies targeting renal outcomes as primary end-points^8,9^. Metformin, a biguanide derivative, is a preferred oral hypoglycemic agent (OHA) to manage T2DM. It is widely introduced as the first-line treatment agent in latest T2DM management guidelines^10,11^. A UKPDS study has shown that metformin treatment in patients with T2DM is associated with a tendentially lower risk of all-cause mortality and major cardiovascular events compared with placebo^12^.

Although metformin is widely used in patients with T2DM, its effect on patients with advanced chronic kidney disease (CKD) is unclear. Lack of these evidence is partially attributed to the fact that metformin is contraindicated in patients with decreased renal function due to concerns of lactic acidosis, a rare but serious complication of metformin. Metformin-associated lactic acidosis has been controversial in its incidence and severity. Investigators have demonstrated that risk of lactic acidosis in metformin users is minimal or non-fatal^13,14^. Based on these clinical evidence, the U.S. Food and Drug Administration (FDA) has changed the recommended contraindication to renal function estimated glomerular filtration rate (eGFR) > 30 ml/min/1.73 m^2^ in patients using metformin^15^. However, there are merely no recent trials comparing effects of metformin with newer OHAs^16^. Given the seriousness of DN, it is important to obtain novel evidence for effects of metformin on cardiovascular events and renal disease progression. Thus, we conducted a multicenter observational study among heterogeneous tertiary hospitals in South Korea to evaluate the effect of metformin on DN using the common data model (CDM).

## Methods

This retrospective, multicentric, observational cohort study using common data model was supported by Observational Health Data Sciences and Informatics (OHDSI) collaborative. The OHDSI has been providing database software based on Observational Medical Outcomes Partnership (OMOP) CDM^17^. We used data from three referral hospitals’ electronic medical records (EMR) databases: Seoul National University Bundang Hospital (SNUBH), Asan Medical center (AMC), and Seoul National University Hospital (SNUH). All three participated institutions are large medical centers located in urban area of South Korea. The EMR of SNUBH, the oldest database, consisted of data from 2003 to 2019. Other two databases (AMC and SNUH) had complete EMR data from 2004 to 2019. All EMR data from each hospital were transformed into OMOP-CDM version 5 schema^18^. The OMOP-CDM database comprised automatically deidentified EMR data including prescriptions, laboratory test, diagnoses, procedures, and demographics of visiting patients. The CDM schema enables identical data analysis execution on heterogenous EMR database using transformed standardized vocabulary.

### Study design and cohort construction

This retrospective, multicenter, observational study employed a comparative cohort design to assess effects of metformin on different groups based on various stages of renal function status. We identified patients with T2DM in each site using OMOP-CDM diagnosis codes. We then cross-validated them through HbA1c level ≥ 6.5% or fasting blood glucose ≥ 126 mg/dL to prevent over aggregation of study subjects. We defined index date (start of observation period) as the visit date of the first diagnosis of T2DM and end date of observation as the last time the study subject visited the hospital. We excluded patients who met the following condition: 1) a diagnosis of type 1 diabetes, 2) an age of less than 18 or over 80 at the index date, 3) prior exposure to corticosteroids for more than 12 weeks or calcineurin inhibitors, which could cause drug-induced diabetes, 4) a history of malignancies, ESKD, myocardial infarction, or cerebral infarction, and 5) had not prescribed any antidiabetic agent six months before or after the index date.

We identified cohorts as follows. First, T2DM cohorts were identified using a specific combination of laboratory tests and diagnosis code to identify diabetic patients who had not yet developed DN at the index date. Second, T2DM with each CKD stage (3A, 3B, and 4) cohort was identified by a combination of prior T2DM cohort definitions and renal function test using eGFR by creatinine-based EPI-CKD equation^19^.

**Figure 1:**
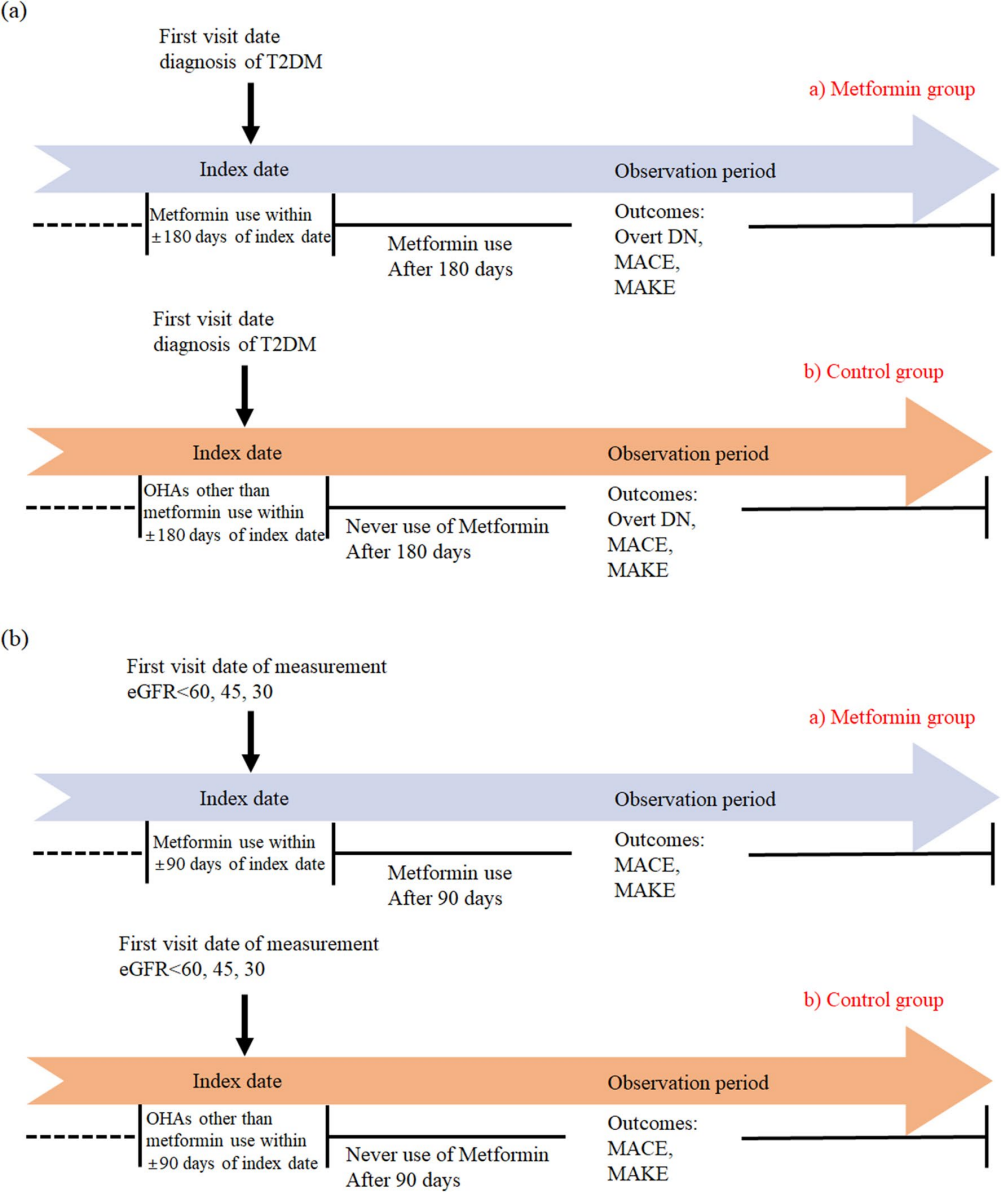
Visual explanations of cohort definition. (**a**) Observational cohort definition of newly diagnosed patients with type 2 diabetes excluding DN at baseline. (**b**) Observational cohort definition of patients with type 2 diabetes and reduced renal function. T2DM: type 2 diabetes, DN: diabetic nephropathy, MACE: major adverse cardiovascular event, MAKE: major adverse kidney event, OHAs: oral hypoglycemic agents.

To define the control cohort for investigating the effect of metformin on DN in patients newly diagnosed with T2DM, we applied the following approach within the common data model1. First, the index date was identified as the first hospital visit date which a diagnosis of T2DM was confirmed via diagnosis codes of ICD-10 and HbA1c level. Second, considering standard clinical practice, patients prescribed metformin in the 180 days surrounding the index date (both prior and subsequent) who continued metformin treatment at least once beyond the initial 180 days were designated as the metformin-using group. Controls were patients who were prescribed other OHAs within the 180-day window surrounding the index date without receiving any further metformin prescriptions after this 180-day period throughout the observation period. Visual explanations of the study cohorts are shown in Fig. 1(a).

To analyze outcomes in T2DM patients with eGFR levels less than 60, 45, and 30 ml/min/1.73 m^2^, respectively, control cohorts were defined in a similar manner. For a patient diagnosed with T2DM (as determined by a specific diagnosis code and HbA1c level) who had two or more confirmed readings of eGFR less than 60, 45, or 30, the index date was established as the time of the first eGFR measurement that fell below the respective threshold. Considering conventional clinical practice for DN patients showing diminished renal function, the metformin user group was defined as those who were prescribed metformin in the 90-day period both prior to and after the index date and continued the use of metformin at least once beyond this 90-day period. The control group consisted of patients who were prescribed OHAs within the 90-day timeframe surrounding the index date without receiving any further metformin prescriptions after this 90-day period during the observation period. Visual explanations of study cohorts are shown in Fig. 1b. After identifying cohorts, four sets of treatment–control comparative cohorts were established within each participating institution. This stratification was designed to elucidate influence of metformin on diverse groups categorized according to respective renal function status.

### Outcomes

Primary outcomes of this study were net major adverse cardiovascular events (MACE) or in-hospital death and a composite of major adverse kidney events (MAKE) or in-hospital death. MACEs were defined as ischemic events including myocardial infarction and ischemic stroke as determined by diagnostic history. MAKE were defined as the need for renal replacement therapy (RRT) or an eGFR of less than 15 ml/min/1.73 m^2^ for more than two consecutive measurements based on the EPI-CKD equation. In addition, this study evaluated the incidence of new-onset DN or overt DN in the T2DM without DN cohort. We defined DN as either spot urine albumin-to-creatinine ratio of 30 mg/g or 24-h albuminuria of 30 mg or more, either spot urine protein-to-creatinine ratio of 150 mg/g or 24-h proteinuria of 150 mg or more, or EPI-CKD eGFR less than 60 ml/min/1.73 m^2^. Overt DN definition was either spot urine albumin-to-creatinine ratio of 300 mg/g or 24-h albuminuria of 300 mg or more, either spot urine protein-to-creatinine ratio of 500 mg/g or 24-h proteinuria of 500 mg or more, or EPI-CKD eGFR less than 60 ml/min/1.73 m^2^.

### Statistical analysis

We used a 1:1 matching of propensity score (PS) to reduce selection bias due to differences between treatment and control groups. PS was estimated by L1 logistic regression adjusted by tenfold cross validation with a caliper of 0.2 on the logit scale. The Cyclops R package (https://github.com/ohdsi/cyclops) was used for PS matching. We used sex, age groups, drug prescriptions other than the target drug, metformin, and disease diagnosis before the index date as covariates for the matching. Measurements and conditions after the index date were not used for matching. We used the Cox proportional hazard model to analyze matched cohorts and calculate hazard ratios (HR) along with 95% confidence intervals (CI) for outcomes. Survival curves were constructed and compared using Kaplan–Meier estimates and log-rank tests. We then conducted meta-analysis to calculate pooled estimated HRs across the three databases. Meta-analysis was conducted using a random-effect model with heterogeneity analysis with *I*^2^ index^20^. To assess robustness, components of outcome such as sensitivity were analyzed^21^. All statistical tests were performed using R (version 3.6.3; the R foundation for Statistical Computing), including packages provided by the OHDSI collaboration^22^.

### Ethical approval

This study was approved by the ethics committee of each institution, and the requirement for informed consent was waived by the Institutional Review Board of Seoul National University Bundang Hospital (IRB No. X-1808–484-906). This study met the criteria for waiver of informed consent as stated in the Bioethics and Safety Act of the Republic of Korea, and all methods used were performed according to the relevant guidelines and regulations.

### Ethics approval and consent to participate

The study was approved by the research ethics committees of the participating centers: The Institutional Review Board of Seoul National University Bundang Hospital, Asan Medical center, and Seoul National University.

## Results

### Clinical outcomes in T2DM without DN cohort

For SNUBH, this study identified 9,023 individuals who had continuously received metformin and 608 individuals who had never been prescribed metformin in the last six months after the index date. At SNUH, this study identified 8,884 individuals who had continuously received metformin and 759 individuals who had never been prescribed metformin for six months after the index date. At AMC, this study used 8,453 individuals in the treatment group and 714 individuals in the control group to construct a treatment–control comparative cohort set. Baseline characteristics after 1:1 PS matching is presented in Table 1. Post propensity score matching, disparities in age, clinical parameters including EPI-CKD eGFR, and underlying medication usage were considerably mitigated.

**Table 1:** Baseline characteristics and medication usage in the T2DM without DN cohort after 1:1 propensity matching.

	SNUBH			AMC			SNUH		
	Metformin cohort	Control cohort	*p-*value	Metformin cohort	Control cohort	*p-*value	Metformin cohort	Control cohort	*p-*value
N	587	587		669	669		738	738	
Sex, female, n (%)	268 (46)	272 (46)	0.81	266 (40)	265 (40)	0.96	319 (0.43)	314 (0.43)	0.79
Age, mean, years (IQR)	65 (55–72)	65 (56–72)	0.93	61 (53–69)	62 (55–69)	0.14	63 (55–70)	64 (56–71)	0.11
Medication use, n (%)									
RAS inhibitors	239 (40.72)	202 (34.41)	0.03	219 (32.74)	218 (32.59)	0.95	257 (34.82)	219 (29.67)	0.03
Beta-blockers	45 (7.67)	62 (10.56)	0.08	91 (13.6)	101 (15.1)	0.44	97 (13.14)	100 (13.55)	0.82
DHP CCB	130 (22.15)	117 (19.93)	0.35	133 (19.88)	125 (18.68)	0.58	147 (19.92)	126 (17.07)	0.16
Non-DHP CCB	6 (1.02)	10 (1.7)	0.31	32 (4.78)	34 (5.08)	0.80	28 (3.79)	39 (5.28)	0.17
Thiazides	79 (13.46)	73 (12.44)	0.60	35 (5.23)	33 (4.93)	0.80	76 (10.3)	62 (8.4)	0.21
Alpha-blockers	17 (2.9)	44 (7.5)	< 0.01	28 (4.19)	19 (2.84)	0.18	19 (2.57)	33 (4.47)	0.05
Statins	203 (34.58)	186 (31.69)	0.29	269 (40.21)	267 (39.91)	0.91	287 (38.89)	283 (38.35)	0.83
Anti-hyperglycemic agent use, n (%)									
Metformin	482 (82.11)	88 (14.99)	< 0.01	543 (81.17)	114 (17.04)	< 0.01	585 (79.27)	95 (12.87)	< 0.01
Sulfonylurea	307 (52.3)	309 (52.64)	0.91	282 (42.15)	326 (48.73)	0.02	307 (41.6)	374 (50.68)	< 0.01
Thiazolidinediones	51 (8.69)	73 (12.44)	0.04	39 (5.83)	45 (6.73)	0.50	43 (5.83)	48 (6.5)	0.59
DPP-IV inhibitors	95 (16.18)	74 (12.61)	0.08	210 (31.39)	223 (33.33)	0.45	161 (21.82)	150 (20.33)	0.48
SGLT-2 inhibitors	10 (1.7)	5 (0.85)	0.19	10 (1.49)	23 (3.44)	0.02	15 (2.03)	20 (2.71)	0.39
Alpha-glucosidase	38 (6.47)	104 (17.72)	< 0.01	54 (8.07)	71 (10.61)	0.11	31 (4.2)	63 (8.54)	< 0.01
GLP-1 agonists	1 (0.17)	1 (0.17)	1.00	0 (0)	0 (0)	NA	1 (0.14)	1 (0.14)	1.00
Insulin	15 (2.56)	15 (2.56)	1.00	0 (0)	0 (0)	NA	13 (1.76)	8 (1.08)	0.27

**Figure 2:**
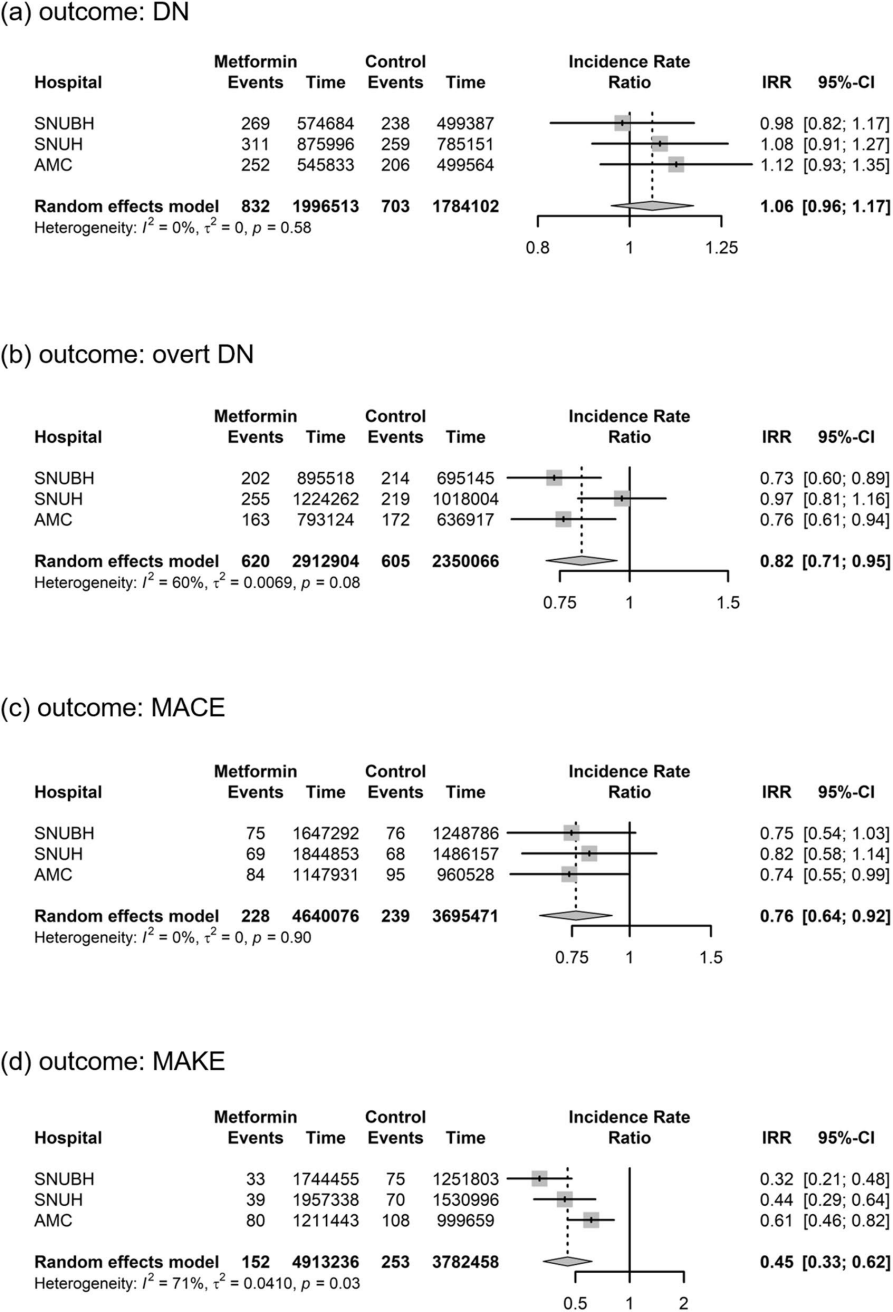
Forest plots showing incidence rate ratio (IRR) with 95% confidence intervals and summarized IRR for T2DM cohorts without DN. Each plot represents meta-analysis outcome of (**a**) DN, (**b**) overt DN, (**c**) MACE, and (**d**) MAKE. T2DM: type 2 diabetes, DN: diabetic nephropathy, MACE: major adverse cardiovascular event, MAKE: major adverse kidney event.

Summarized incidence rate ratios (IRRs) of DN, overt DN, MACE and MAKE between matched cohorts were found to be 1.06 (95% CI 0.96–1.17), 0.82 (95% CI 0.71–0.95), 0.76 (95% CI 0.64–0.92), and 0.45 (95% CI 0.33–0.62), respectively (Fig. 2).

### Clinical outcomes in T2DM with CKD3A, CKD3B, and CKD4 cohort

**Figure 3:**
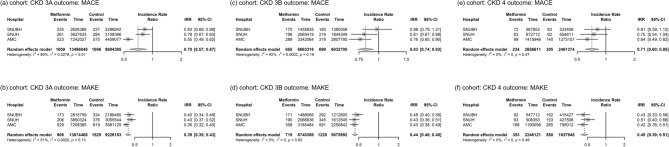
Forest plots showing incidence rate ratio (IRR) with 95% confidence intervals and summarized IRR for T2DM cohorts having renal function of CKD stages 3A, 3B, and 4. Each plot represents meta-analysis outcome of (**a**) MACE in patients with CKD 3A, (**b**) MAKE in patients with CKD 3A, (**c**) MACE in patients with CKD 3B, (**d**) MAKE in patients with CKD 3B, (**e**) MACE in patients with CKD 4, and (**f**) MAKE in patients with CKD 4. T2DM: type 2 diabetes, CKD: chronic kidney disease, MACE: major adverse cardiovascular event, MAKE: major adverse kidney event.

The study population consisted of individuals who had either continuously received metformin treatment or had not been prescribed metformin for three months after the index date, at the start of each respective CKD stage. At SNUBH, this study identified 4,709 individuals with CKD3A, 2,003 individuals with CKD3B, and 579 individuals with CKD4 in the treatment group and 1,595 individuals with CKD3A, 1,244 individuals with CKD3B, and 997 individuals with CKD4 in the control group. At SUNH, we found 6,169 individuals with CKD3A, 2,552 individuals with CKD3B, and 650 individuals with CKD4 in the treatment group, and 2,314 individuals with CKD3A, 1,833 individuals with CKD3B, and 1,428 individuals with CKD4 in the control group. At AMC, we found 6,830 individuals with CKD3A, 2,976 individuals with CKD3B, and 1043 individuals with CKD4 in the treatment group and 3,221 individuals with CKD3A, 2,538 individuals with CKD3B, and 1,912 individuals with CKD4 in the control group. We found that summarized incidence rate ratios (IRRs) of MACEs and MAKEs were lower in the matched cohorts with CKD3A, CKD3B, and CKD4. For CKD3A, summarized IRRs of MACEs and MAKEs were 0.70 (95% CI 0.57–0.87) and 0.39 (95% CI 0.35–0.43), respectively. For CKD3B, summarized IRRs of MACEs and MAKEs were 0.83 (95% CI 0.74–0.93) and 0.44 (95% CI 0.40–0.48), respectively. For CKD4, summarized IRRs of MACEs and MAKEs were 0.71 (95% CI 0.60–0.85) and 0.45 (95% CI 0.39–0.51), respectively (Fig. 3).

### Clinical parameters during observation period

We additionally measured median values of clinical parameters and medication use during the observation period in the T2DM without DN cohort. Across all three institutions, the median HbA1c level in the metformin cohort was significantly higher than that in the control cohort. The EPI-CKD eGFR was revealed to be consistently higher in the metformin group than in the control group (Table 2).

**Table 2:** Clinical parameters during the observation period in the T2DM without DN cohort after 1:1 propensity matching.

Clinical parameters	SNUBH			AMC			SNUH		
	Metformin cohort	Control cohort	*p-*value	Metformin cohort	Control cohort	*p-*value	Metformin cohort	Control cohort	*p-*value
N	587	587		669	669		738	738	
HbA1c, % (IQR)	7.2 (6.7–7.8)	6.8 (6.4–7.5)	< 0.01	7.3 (6.8–8.1)	7.0 (6.5–8.1)	< 0.01	7.2 (6.6–7.8)	6.9 (6.4–7.6)	< 0.01
Serum creatinine, mg/d (IQR)	0.9 (0.7–1.0)	0.9 (0.8–1.1)	< 0.01	0.8 (0.7–0.9)	0.8 (0.7–1.0)	0.01	0.9 (0.8–1.0)	0.9 (0.8–1.1)	0.04
EPI-CKD eGFR, ml/min/1.73 m^2^ (IQR)	86.1 (70.6–98.3)	80.8 (63.5–95.5)	< 0.01	96.8 (83.3–107.2)	93.5 (75.8–105.8)	< 0.01	85.0 (69.7–97.7)	81.5 (67.2–96.2)	0.01
Urine albumin/creatinine ratio, mg/g(IQR)	16 (9–39)	15 (8–44)	0.70	18 (8–65)	13 (6–76)	0.02	15 (8–67)	13 (6–72)	0.10
Urine protein/creatinine ratio, mg/g (IQR)	136 (92–282)	195 (107–1125)	< 0.01	NA	NA	NA	149 (84–470)	185 (92–951)	0.04
Hemoglobin, g/dL(IQR)	13.2 (11.9–14.5)	13.0 (11.9–14.2)	0.09	12.9 (12.2–14.0)	12.7 (11.5–14.2)	0.05	13.4 (12.1–14.6)	13.2 (12.1–14.5)	0.20
Uric acid, mg/dL (IQR)	5.0 (4.2–6.0)	5.1 (4.2–6.2)	0.20	4.8 (4.1–5.8)	4.8 (3.9–5.9)	0.66	5.1 (4.2–6.1)	4.9 (4.0–5.6)	0.01
Phosphate, mg/dL (IQR)	3.5 (3.3–3.8)	3.6 (3.3–3.8)	0.69	3.4 (3.1–3.8)	3.5 (3.1–3.9)	0.04	3.5 (3.3–3.8)	3.5 (3.2–3.8)	0.01
Potassium, mmol/L (IQR)	4.3 (4.1–4.5)	4.3 (4.1–4.6)	0.97	4.3 (4.1–4.4)	4.2 (4.1–4.5)	0.91	4.4 (4.2–4.6)	4.3 (4.1–4.6)	0.02
tCO2, mmol/L (IQR)	26 (25–27)	26 (24–27)	0.11	25 (24–27)	25 (24–27)	0.22	27 (25–29)	27 (25–29)	0.10
Albumin, g/L (IQR)	4.3 (4.1–4.4)	4.2 (3.9–4.4)	< 0.01	3.9 (3.6–4.2)	3.8 (3.4–4.1)	< 0.01	4.3 (4.1–4.5)	4.2 (3.9–4.4)	< 0.01
Alkaline phosphate, IU/L (IQR)	73 (63–89)	76 (62–95)	0.04	71 (60–89)	77 (63–102)	< 0.01	66 (57–79)	71 (59–87)	< 0.01
Cholesterol, mg/dL (IQR)	165 (150–180)	170 (151–189)	0.01	151 (132–170)	159 (134–177)	< 0.01	159 (141–177)	161 (143–182)	0.04
Intact-PTH, pg/mL (IQR)	29.0 (23.0–38.9)	33.2 (22.0–46.1)	0.14	31.0 (20.8–48.5)	37.3 (22.4–70.3)	0.20	31.4 (21.3–58.9)	29.5 (17.8–42.8)	0.50

### Sub-outcome analysis

To elucidate the influence of metformin on each component contributing to the primary composite outcome, we conducted sub-outcome analysis^23^. For the development of DN, albuminuria and eGFR below 60 ml/min/1.73 m^2^ showed comparable frequencies, whereas proteinuria contributed to a lesser extent. In-hospital mortality, a component of MAKE and MACE, occurred with similar frequency in metformin and control cohort comparisons. However, a diminished risk was observed in the metformin group for renal outcome of eGFR below 15 ml/min/1.73 m^2^.

## Discussion

In our retrospective, observational cohort study across databases of three tertiary hospitals in Korea, continuously use of metformin in patients with newly diagnosed T2DM compared with the control cohort was associated with lower risks of overt DN, MACEs, and MAKEs. There was no significant difference in the risk of event of DNs (including urine albumin-to-creatinine ratio > 30 mg/g and urine protein-to-creatinine ratio > 150 mg/g). Findings for each CKD stage (3A, 3B, and 4) revealed that continuously use of metformin compared with non-metformin user or discontinued user cohort was associated with low risks of MACEs and MAKEs. To our best knowledge, this represents the first effort to distinctly illustrate long-term renal and cardiovascular outcomes corresponding to distinct renal function states in a comprehensive, multicenter observational study.

Metformin is a commonly used anti-diabetic drug that has been in clinical use for over 60 years. Metformin is selected as the primary therapeutic intervention for T2DM attributable to its demonstrated efficacy, low cost, and commendable safety profile^24^. However, metformin use in patients with renal function below 30 ml/min/1.73 m^2^ has been contraindicated due to concerns over its potential to cause lactic acidosis, a rare but potentially fatal side effect^25^. Despite concern of adverse effect, several studies have proposed that metformin could confer potential benefits for diabetic patients exhibiting diminished renal function. An observational, national cohort study encompassing 175,296 novel metformin or sulfonylurea monotherapy users has found that metformin monotherapy is correlated with a lower risk of all-cause mortality, with those in the CKD3B category (eGFR 30 to 44 ml/min/1.73 m^2^) manifesting the greatest absolute risk reduction, ranging from − 19.0 to − 5.2 deaths per 1000 person-years^26^. A post-hoc analysis of the Trial to Reduce Cardiovascular Events with Aranesp Therapy (TREAT trial) focusing on diabetic patients with CKD has revealed a correlation between baseline metformin use and lower risks of all-cause mortality, cardiovascular events, and composite renal outcomes (ESKD and death). Nonetheless, associations with ESKD and doubling of serum creatinine levels did not exhibit statistical significance^27^. In a meta-analysis of all-cause mortality and cardiovascular events in patients with coexisting CKD and T2DM, metformin-based treatments were associated with significant lower risks of all-cause mortality and cardiovascular events in type 2 diabetic patients with CKD stages 1 to 3. However, the same meta-analysis did not delineate a significant impact in all-cause mortality or cardiovascular events in patients with stage 4 or 5 CKD^28^. Previous studies have assessed pharmacological stability and determined the optimal dosing by measuring trough levels of metformin and concentrations of lactic acid in patients with T2DM exhibiting diminished renal function. They affirmed that a daily dosage of 500 mg per day of metformin maintained minimal serum and erythrocyte metformin concentrations without inducing hyperlactatemia exceeding 5.0 mmol/L in patients with CKD4^29,30^.

Considering these recent findings for metformin as an old drug, compelling real-world evidence is required to advocate for application of metformin in patients with advanced CKD. The present study organized data originating from multiple, equivalently sized but diverse centers, each representing various stages of renal function, from without DN to stage 4 CKD. The utilization of real-world multicentric EMR data is increasingly emerging as an alternative method to address hurdles associated with randomized clinical trials^31^. These programs can facilitate the transformation of data from diverse databases with OMOP-CDM standardized vocabularies. They function as tools for observational cohort studies, facilitating comparative analysis and mitigating biases through PS matching^17,22,32^.

While our findings offer valuable insights, we acknowledge that our study has certain limitations. Considering the retrospective, observational nature of our study, the possibility of selection bias cannot be disregarded. To counteract this, we endeavored to conduct PS matching on various confounding variables by leveraging a comprehensive observational dataset comprised of a large patient cohort. Nevertheless, during cohort generation, selection bias might have been introduced due to inherent limitations presented by a retrospective real-world setting study. Additionally, despite our efforts of a 1:1 PS matching to counterbalance confounding factors, several variables such as serum albumin and HbA1c levels remained unbalanced after matching. This imbalance could potentially introduce a bias when interpreting study findings. For example, the relatively high HbA1c level in the metformin groups may have been itself associated with unfavorable prognosis and may have had a negative impact on hard outcomes such as in-hospital death. Considering the inherent limitations associated with retrospective observational studies, further well-designed randomized controlled trial for metformin administration is imperative. However, the significance of the current study is highlighted by its integration of real-world clinical data, which encompasses patients diagnosed with CKD4 (eGFR 15 to 29 ml/min/1.73 m^2^), a group currently considered contraindicated.

## Conclusions

Using a common data model from database of disparate electronic health records, our findings suggest that administering metformin in patients with T2DM is associated with a lower risk of overt diabetic nephropathy and major cardiovascular events. Furthermore, for patients with T2DM who have reduced renal functions, continuing metformin treatment is associated with lower risks of major kidney and cardiovascular events. Our study is a multicentric observational cohort study that employs a common data model. By utilizing this standardized study methodology, we can examine robust real-world evidence for effects of various treatments employed in clinical practice for patients with T2DM.

## Data Availability

Individual participant data will not be available. Documents available: study protocol, statistical analysis plan, and analytic code. Data is to be used for approved proposal aims only. Send data access requests to yongjin.neph@gmail.com and sign a data access agreement.

## Abbreviations

T2DM: Type 2 diabetes mellitus
OHA: Oral hypoglycemic agent
DN: Diabetic nephropathy
ESKD: End-stage kidney disease
CKD: Chronic kidney disease
eGFR: Estimated glomerular filtration rate
OHDSI: The Observational Health Data Sciences and Informatics
OMOP: Observational Medical Outcomes Partnership
CDM: Common data model
EMR: Electronic medical records
MACE: Major adverse cardiovascular events
MAKE: Major adverse kidney events
RRT: Renal replacement therapy
PS: Propensity score
CI: Confidence interval
HR: Hazard ratio
IRR: Incidence rate ratios
