# Author Correction: Impact of metformin on cardiovascular and kidney outcome based on kidney function status in type 2 diabetic patients: a multicentric, retrospective cohort study

**DOI:** 10.1038/s41598-024-62989-x

**Published:** 2024-05-29

**Authors:** Yongjin Yi, Eun‑Jeong Kwon, Giae Yun, Seokwoo Park, Jong Cheol Jeong, Ki Young Na, Ho Jun Chin, Sooyoung Yoo, Seok Kim, Tae Jung Oh, Sejoong Kim, Chang Hee Jung, Hajeong Lee

**Affiliations:** 1 Department of Internal Medicine, Dankook University College of Medicine, Cheonan-Si, Republic of Korea; 2 Division of Nephology, Department of Internal Medicine, Seoul National University Bundang Hospital, Seongnam-Si, Republic of Korea; 3 Healthcare ICT Research Center, Seoul National University Bundang Hospital, Seongnam-Si, Republic of Korea; 4 Department of Internal Medicine, Seoul National University College of Medicine, Seoul, Republic of Korea; 5 Center for Artifcial Intelligence in Healthcare, Seoul National University Bundang Hospital, Seongnam-Si, Republic of Korea; 6 Department of Endocrinology and Metabolism, Asan Medical Center, University of Ulsan College of Medicine, Seoul, Korea; 7 Department of Internal Medicine, Seoul National University College of Medicine, Seoul, Korea; 8 Department of Internal Medicine, Seoul National University Hospital, Seoul, Korea

Correction to: *Scientific Reports* 10.1038/s41598-024-52078-4, published online 24 January 2024

Chang Hee Jung and Hajeong Lee were omitted from the author list in the original version of this Article.

Their affiliations are listed below:

Department of Endocrinology and Metabolism, Asan Medical Center, University of Ulsan College of Medicine, Seoul, Korea

Department of Internal Medicine, Seoul National University College of Medicine

Department of Internal Medicine, Seoul National University Hospital, Seoul, Korea

As a result the author contribution section now reads:

YY interpreted the data, wrote the manuscript, and generated the associated figures and tables. SK and SY managed CDM database and analyzed the data. TJO organized the list OHAs in CDM. CHJ and HL contributed to the data collection and integration into the common data model. EJK, GAY, SP, JCJ, KYN, HJC, and SK contributed to the interpretation and discussion, and reviewed and edited the manuscript. All authors have read and approved the manuscript.

The original Article has been corrected.

